# Calibration of rotation axes for multi-axis goniometers in macromolecular crystallography

**DOI:** 10.1107/S1600576718010956

**Published:** 2018-09-13

**Authors:** K. Ian White, Valeria Bugris, Andrew A. McCarthy, Raimond B. G. Ravelli, Krisztián Csankó, Alberto Cassetta, Sandor Brockhauser

**Affiliations:** a Department of Molecular and Cellular Physiology, Stanford University, Campus Drive, Stanford, CA 94305, USA; b European Molecular Biology Laboratory, Grenoble Outstation, 71 avenue des Martyrs, Grenoble, 38042, France; c Howard Hughes Medical Institute, Stanford University, Stanford, CA 94305, USA; d Biological Research Centre (BRC), Hungarian Academy of Sciences, Temesvári körút 62, Szeged, Csongrad 6726, Hungary; e M4I Division of Nanoscopy, Maastricht University, PO Box 616, MD Maastricht, 6200, The Netherlands; f XRD1 Beamline – Elettra, CNR – Istituto di Cristallografia – Unità di Trieste, S.S. 14 Km 163,5, Trieste, Basovizza I-34012, Italy; g European X-ray Free-Electron Laser Facility GmbH (XFEL.EU), Holzkoppel 4, Hamburg, Schenefeld 22869, Germany

**Keywords:** kappa diffractometer, macromolecular crystallography, reorientation, calibration

## Abstract

The installation of multi-axis goniometers such as the ESRF/EMBL miniKappa goniometer system has allowed the increased use of sample reorientation in macromolecular crystallography. Old and newly appearing data collection methods require precision and accuracy in crystal reorientation. The proper use of such multi-axis systems has necessitated the development of rapid and easy to perform methods for establishing and evaluating device calibration. A new diffraction-based method meeting these criteria has been developed for the calibration of the motors responsible for rotational motion. This method takes advantage of crystal symmetry by comparing the orientations of a sample rotated about a given axis and checking that the magnitude of the real rotation fits the calculated angle between these two orientations. Hence, the accuracy and precision of rotational motion can be assessed. This rotation calibration procedure has been performed on several beamlines at the ESRF and other synchrotrons. Some resulting data are presented here for reference.

## Abstract 2

An easy to perform rotation calibration procedure has been developed for miniKappa and/or other multi-axis goniometers used in macromolecular crystallography to enhance the precision of experiments involving crystal reorientations.

## 1. Introduction   

Exactly 50 years after the kappa patent granted to Nonius (Poot, 1968 ▸), we can state that, although multi-axis goniometers have long been exploited in the realm of small-molecule crystallography, their size as well as implementation challenges at user-oriented facilities has limited their adoption by the macromolecular crystallography (MX) community. A renewed focus on miniaturization has led to the development of devices that integrate seamlessly with many current sample environments at synchrotrons (McCarthy *et al.*, 2009 ▸; Brockhauser *et al.*, 2011 ▸, 2013 ▸; Waltersperger *et al.*, 2015 ▸; Mueller-Dieckmann *et al.*, 2015 ▸; Grama & Wagner, 2017 ▸). By greatly expanding the range over which a given sample can be reoriented, automated multi-axis goniometer systems provide additional freedom and convenience in the optimal design of diffraction experiments.

A major problem in MX continues to be radiation damage (Hendrickson, 1991 ▸; Zeldin *et al.*, 2013 ▸), and it has been a major driving factor behind a great deal of innovation owing to its role in undermining MAD (multi-wavelength anomalous dispersion) or SAD (single-wavelegth anomalous dispersion) phasing experiments (Ravelli *et al.*, 2005 ▸). The inverse-beam method has historically proven useful in mitigating this issue (Dauter, 1997 ▸; Ravelli *et al.*, 1997 ▸). Unfortunately, a number of challenges associated with this method often render it ineffective. For example, many seconds may be required to rotate and launch a data acquisition every half turn. Furthermore, in the case of room-temperature data collection, where the amount of collectable isomorphous data from a single crystal is strongly limited by radiation damage, such a frequent and rapid acceleration can cause the sample to move relative to the original. In all such cases, the resulting data will be more susceptible to errors related to crystal misalignment or poor spindle synchronization.

The alternative solution made possible by a multi-axis goniometer is quite elegant in comparison. The even-fold (2×, 4×, 6×) symmetry axis of the crystal can rapidly be aligned with the spindle to record Bijvoet pairs (a reflection and the Friedel pair of its symmetry equivalent, *i.e.* *hkl* and 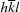) on the same diffraction image. This optimized data collection strategy reduces radiation damage occurring between recording the reflection pairs used for measuring the anomalous differences, and eliminates technical issues related to constantly rotating the sample back and forth at high speed. This method can also be performed manually on single-axis goniometers or non-automated multi-axis instruments, but limitations in available rotational degrees of freedom prevent its usefulness because of difficulties related to properly aligning the twofold symmetry axis of a sample along the spindle axis (Dauter, 1999 ▸).

Another symmetry-related advantage of the alignment of a crystal along specific crystallographic axes relates to the optimization of dose required for point group and space group determination. Applications such as *POINTLESS* (Evans, 2006 ▸) and *AIMLESS* (Evans & Murshudov, 2013 ▸) are capable of determining the space group of a sample on the basis of diffraction statistics such as the presence of systematic absences. Simply reorienting the crystal to measure these reflections can enable the experimental determination of the space group directly.

One of the most common problems for macromolecular complex structures is their tendency to form crystals with large unit-cell dimensions. While dependent on crystal morphology and mounting processes, it is common to encounter some rotation angles with reflection overlap when using the oscillation method. Although an adequate rotation range per single exposure can be determined, it is often too small for even a fine-slicing approach, especially for CCD-based X-ray detectors. Fortunately, the maximum usable range is also governed by the length of the primitive unit-cell dimension along the direction of the X-ray beam and can be optimized by aligning the densest reciprocal space axis (usually the c* crystallographic axis) along the spindle. Note that such a cell alignment results in a blind zone (Dauter, 1999 ▸), which does not allow the collection of a full data set in that orientation. Finding a slightly tilted alignment where the full data set can be collected while the long axis approaches the spindle as much as possible is a good compromise, but such precise realignment is only possible in many cases with a multi-axis system. A less elegant but commonly used alternative is to manually bend the pins, but such an approach is not optimal and cannot be automated.

The crystal-reorientation capabilities of multi-axis goniometers also permit the precise scaling of data collected from multiple crystals or from multiple locations on large single crystals. A data collection protocol can be designed to incorporate diffraction images from multiple crystals oscillated about the same crystallographic axis with exact overlaps.

The greater rotational freedom afforded by multi-axis systems is useful with respect to phasing strategies as well. Bricogne *et al.* (2005 ▸) originally noted substantial dichroism and anisotropy in resonant scattering in X-ray data collected from selenated protein with a brominated inhibitor near the Se and Br *K* edges. The authors subsequently proposed a methodology for optimizing the anomalous phasing signal obtained from SAD or MAD experiments based on crystal alignment relative to the incident beam. Anisotropy in resonant scattering is fundamentally connected to the orientation of a sample’s chemical bonds relative to the polarization of incoming X-radiation, and the information contained therein can be examined prior to any knowledge of the precise locations of scattering sites. However, taking advantage of the polarization anisotropy of anomalous scattering (AAS) requires a motorized multi-axis goniometer in order to reorient the crystal such that the maximum anomalous phasing signal can be attained. Schiltz & Bricogne (2008 ▸) subsequently took these studies a step further and have shown that AAS can also be used to effectively amplify the phasing power in SAD or MAD experiments. This method requires precise knowledge of the orientation of the crystal with respect to the direction of X-ray polarization. The authors subsequently describe the computation of AAS using a multi-axis goniometer setup knowing precisely the direction vectors of the goniometer rotation axes (Schiltz & Bricogne, 2009 ▸). These studies together with improved S-SAD phasing (Olieric *et al.*, 2016 ▸) present compelling arguments for the use of multi-axis goniometers in MX. Other reorientation-based techniques, like flattening CrystalDirect loops (Zander *et al.*, 2015 ▸) or aligning needles for helical scans (Flot *et al.*, 2010 ▸), all rely on the accurate and precise knowledge of the location and direction vectors of the goniometer’s rotational axes.

## 2. Calibration methodology   

### 2.1. Crystal orientation   

Geometrically speaking, the 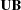 matrix is a product that describes the rotation of a square orthogonalization matrix 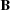 about the square rotation matrix 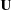 in three dimensions and thus links the coordinates of some vector 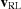 in the reciprocal lattice basis of a crystal to the equivalent vector 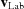 in the laboratory Cartesian system (Busing & Levy, 1967 ▸; Paciorek *et al.*, 1999 ▸; Schiltz & Bricogne, 2009 ▸). As such, some indexed scattering vector 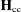 in the Cartesian coordinate system of the crystal can be calculated from its corresponding vector
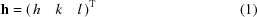
of integer Miller indices and an appropriately calculated orthonormalization matrix 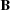 that accounts for crystal symmetry:
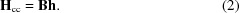


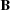
 is typically defined in three dimensions using the convention established by Busing & Levy (1967 ▸), where a* is collinear with the *x* axis of the Cartesian system and **c** is collinear with the *z* axis.

The 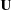 rotation matrix can be decomposed into the sequence of rotations about the three coordinate axes (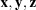) as described by Paciorek *et al.* (1999 ▸), where 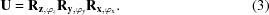


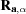
 is the matrix representation of the rotation about the normalized vector **a** by the angle α, and can also be represented in axis–angle form 〈**a**, α〉, in the rotation vector form of 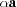 or in the form of a quaternion 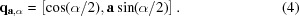
The orthonormal coordinates are then transformed by the rotation matrix such that all of the axes from the original crystal Cartesian system are parallel to the corresponding axes in the laboratory frame of reference when all used goniometer angles are equal to zero. Therefore, 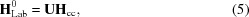
which depends entirely on the fixed laboratory coordinate system chosen. Here, the right-handed Cambridge laboratory frame is used, with the *x* axis along the incident beam, the *z* axis perpendicular to the *x* axis and being in the plane established by the beam and the spindle, and the *y* axis perpendicular to the *x* and *z* axes. Additional conventions followed by different data processing programs have been summarized by Schiltz & Bricogne (2008 ▸).

An additional rotation matrix 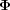 containing geometric information about all rotational goniometer axes is then applied such that 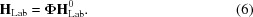



In the case of kappa goniometers, like the multi-axis EMBL/ESRF miniKappa goniometer head, the goniometer rotation 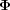 is a product of sequential rotations about the direction vectors of the goniometer axes (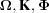) at zero-valued settings by the actual datum applied (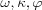), where 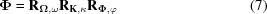
and, in terms of the original variables, 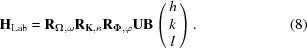
Note that 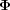 is representing a rotation that is affected by both the angular accuracy of the goniometer and the precision of its axes. The use of an inaccurate 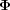, for example during indexing, leads directly to a systematically disturbed 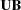 matrix calculation.

A properly calibrated goniometer setup is often difficult to maintain, especially in the context of a system under constant use at an MX beamline. Previous studies have focused on long and laborious methods for initial calibration of such systems (Paciorek *et al.*, 1999 ▸), but no methods currently address the need in MX to rapidly ensure the rotational accuracy of a goniostat. In this paper, a rapid and robust rotation calibration (RC) method is presented. The method takes advantage of the relationship between the two unique sets of vectors 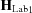 and 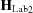 derived from separate indexings of data collected on a given crystal to calculate the angular direction and magnitude of a rotation about any of the axes in a goniometer system. The result of this calculation, when compared to the expected value, yields valuable information regarding the rotational accuracy and precision of the system. Furthermore, recommendations regarding a proper calibration technique will be presented and efforts to integrate such methodology into current data collection systems will be discussed.

### 2.2. Calibration protocol   

Calibration of one of the rotation axes on a multi-axis goniometer requires precise and accurate knowledge of a crystalline sample’s orientation matrix at a reference angle 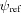 and at some other angle 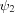. Each axis must be calibrated independently to avoid convolution of its own error with that of the other rotation axes. For calibration of a three-axis κ goniometer with rotation axes (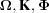), two diffraction data sets are collected per axis from a crystal of known geometry at settings of 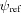 and 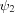 (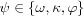) while the other goniometer settings 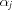 are kept unchanged. The diffraction images are then autoindexed as if they had been collected on a single-axis goniometer with starting spindle position equal to zero, where 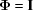, the identity. This yields two 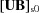 matrices per axis, referred to here as orientation matrices 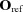 and 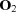, which describe the orientation of the sample’s unit cell in the fixed laboratory coordinate system. Following the multi-axis convention, the orientation matrix can thus be written as 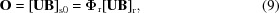
where the single-axis 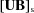 matrix is decomposed into the product of the real 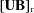 matrix and the real multi-axis reorientation matrix. Note that the matrix 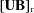 does not depend on the goniometer settings. Hence, in the case of the *i*th axis, where 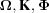 of a κ goniometer are the first, second and third axis, respectively,
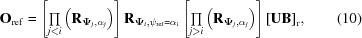


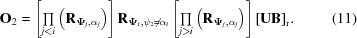



To calculate the angular offset between the two orientations, we assume that there is some transformation matrix 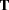 that converts 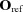 to 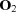, where
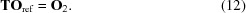
Then, upon rearranging and using the abbreviation
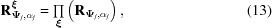


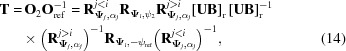
which reduces to 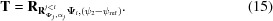
Hence, knowing the direction vectors and the applied angles of all the axes in front of the axis in question, the direction vector of the axis at zero-valued goniometer settings (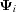) can be calculated by transforming the rotation axis represented by 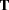 using the inverse of the product (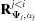). If all the angle settings for the axes in front as well as the reference are kept at zero, the transformation matrix, 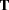, represents a rotation about the directional vector of the axis at zero-valued goniometer settings by the angular difference applied between the two data sets (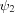).

Owing to possible equivalent indexing of the same lattice, however, multiple solutions for the orientation matrix may exist, where 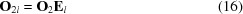
with matrices 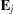 corresponding to equivalent lattice re-indexing transformations. As such, by comparing 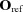 with the set of all possible values of 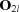, the set of transformation matrices 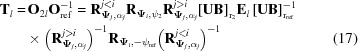
can be determined. Note that 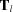 forms a rotation matrix only if the appropriate re-indexing transformation is applied such that 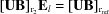.

In practice, however, 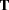 never forms a perfect rotation matrix. 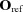 and 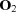 are generated by two different observations and thus two different indexing solutions, and may have differences resulting from both rotational and non-rotational instabilities related to mechanical problems with the motors (*e.g.* slippage or loss of steps) as well as to minor issues with unit-cell determination during auto-indexing. As such, 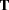 is re-orthogonalized to form a pure rotation matrix 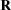, where 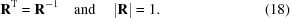
The re-orthagonalization can be performed using the singular value decomposition (SVD) (Golub & Reinsch, 1971 ▸) normalization method, similar to that described by Challis (1995 ▸), although other methods involving the use of quaternions have also been developed to perform such a computation (Bar-Itzhack, 2000 ▸).

The re-orthogonalized 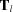 matrices (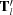) can be determined in axis–angle representation 〈
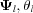
〉: 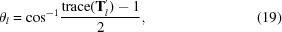


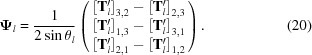
Assuming mostly ideal rotation about the axis in question, the final rotation matrix 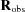 with corresponding axis–angle 〈
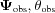
〉 can be found by identifying the observed angle closest to the expected rotation, where 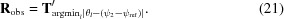
These metrics provide feedback on the precision and angular accuracy of the system by illustrating the agreement between the differences of rotation angles used as goniometer settings for subsequent data collections 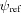 and 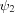 and the calculated rotation angle 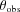.

## 3. Discussion   

For a precise reorientation, accurate information on the direction vectors of the rotation axes is required. In the case of traditional multi-axis goniometers, the manufacturer provides such information and one simply assumes a perfectly aligned instrument. Subsequent readjustment to this level of precision is complex and can require a tremendous amount of work and time. In contrast, the computational calibration solution presented here offers a simple five-step calibration procedure for multi-axis goniometers that can be performed in a matter of minutes if automated as described below.

**Figure 1:**
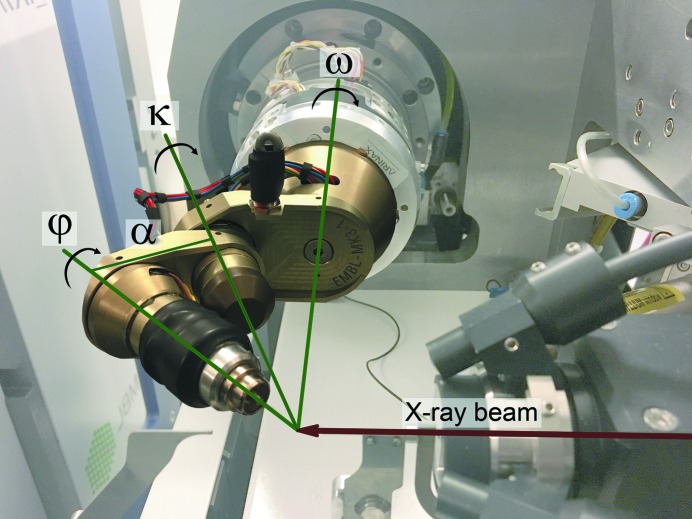
Experiment setup on the ID30B beamline at the ESRF (McCarthy *et al.*, 2018 ▸) with a miniKappa goniometer. The rotation axes are mounted in the following order, Omega (**Ω**), Kappa (**K**) and Phi (**Φ**). The angle alpha (α) between Kappa and Phi is nominally 24° by design. In the captured goniometer settings, Kappa is open by 180°, so Phi and Omega are separated by 2α. A sample is mounted in a loop at the end of a magnetically held pin on the Phi axis for positioning in the X-ray beam.

In practice, calibration takes very little time to perform on a beamline when combined with the software solution *STAC* (strategy for aligned crystals; Brockhauser *et al.*, 2013 ▸), which implements the RC method described here. Such a calibration can be quickly performed for the EMBL/ESRF miniKappa goniometer head (Fig. 1 ▸), or even others, as follows:

(i) After homing the rotation motors and mounting an arbitrary well diffracting single crystal, collect one or more diffraction images about 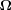 (the same spindle axis used for collecting all the data during the calibration) at a starting angle of 0° for indexing. *STAC* accepts indexing results from *MOSFLM* (Leslie, 2006 ▸), *XDS* (Kabsch, 1988 ▸), *DENZO* (Otwinowski & Minor, 1997 ▸) and the *DNA* expert system (Leslie *et al.*, 2002 ▸).

(ii) Rotate the 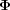-axis motor by a given angle. Collect new image(s), and index.

(iii) Rotate the 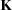-axis motor by a given angle. Collect new image(s), and index.

(iv) Rotate the 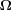 axis by a given angle. Collect new image(s) at this new starting angle, and index.

**Figure 2:**
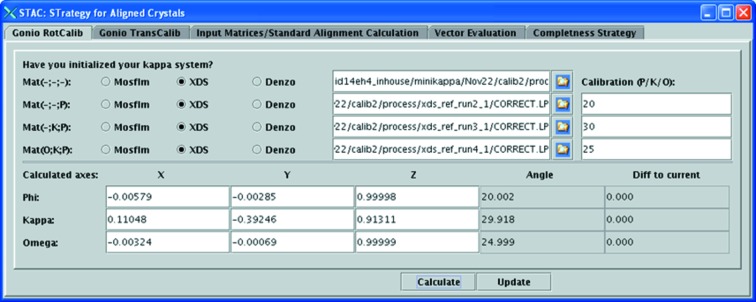
*STAC* graphical user interface facilitating the rotation calibration procedure. *XDS* indexing results from four different goniometer settings [omega(start)–kappa–phi: 0°–0°–0°, 0°–0°–20°, 0°–30°–20°, 25°–30°–20°] are used as input. The results of rotation calibration are presented in axis–angle representation using the Cambridge laboratory frame convention (Powell *et al.*, 2013 ▸), as implemented at the ESRF. Calculated rotation angles show good agreement with the input motor movements requested. Also note that the angular difference between the calculated direction vectors of the Phi and Kappa axes is 23.9966°, which is in a good agreement with the nominal alpha value of the miniKappa design equal to 24°.

(v) Provide the indexing results together with the applied rotation angles to *STAC*, which then computes 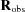 for all the rotation axes and displays them in an axis–angle representation (see Fig. 2 ▸.)

This method assumes that all other components of the beamline are properly calibrated, as it is based on the collection of diffraction images. A well diffracting low-mosaic crystal should be used in order to generate the most accurate orientation matrix possible. Since diffraction images are collected in different orientations, it is important to always expose the same part of the crystal in the X-ray beam, so the diffracting volume does not change between orientations. Where possible, it is best achieved using a beam size matching the crystal size.

**Figure 3:**
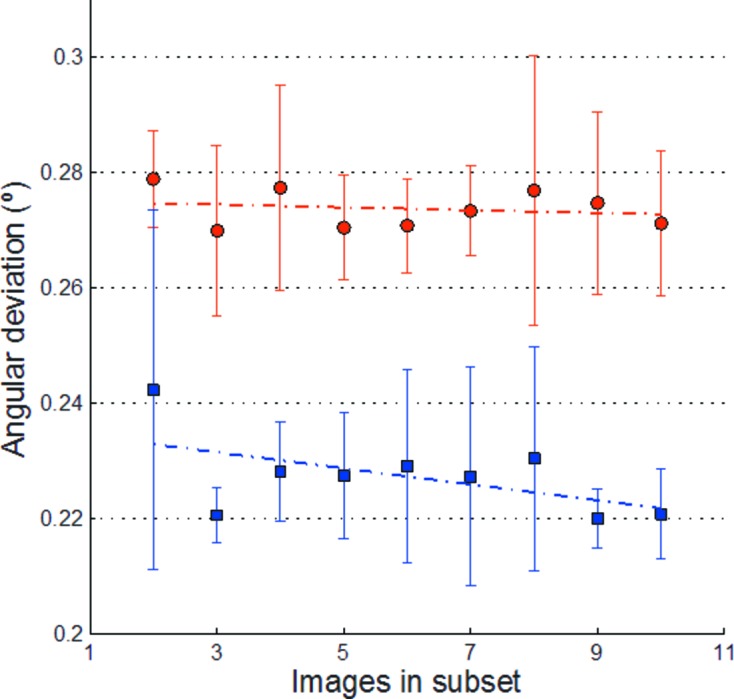
Orientation matrix calculation for two sets of data collected from separate cubic insulin crystals on ID14-4 at the ESRF. Two supersets of 60 images were collected, one with 0.5° wedges (filled circles) and the other 1.0° wedges (filled squares). For each, five subsets of images were randomly generated for sets varying in length from two to ten images. The supersets, and each subset, were processed using *XDS*. The angular deviation difference between the orientation matrix for each subset and the appropriate superset was then calculated The average values and corresponding variances are shown.

A number of variables were tested in order to determine ideal calibration conditions with respect to the calculation of correct orientation matrices and their subsequent use for RC. Hen egg-white lysozyme, trypsin, thaumatin and insulin crystals could all be rapidly auto-processed with *XDS* (Kabsch, 2010 ▸) to produce consistent orientation matrices. Representative results from insulin and thaumatin are presented in Fig. 3 ▸ and Table 1 ▸.

### 3.1. Calibration accuracy   

The presented RC method is based on orientation determination at different goniometer settings. Hence, it is important to characterize the stability of the indexing protocol used.

For this purpose, data were collected from two insulin crystals (Nanao *et al.*, 2005 ▸) on ID14-4 at the ESRF. [Fig. 1 ▸ shows an identical setup on the successor beamline ID30B (McCarthy *et al.*, 2018 ▸), which was also used to produce the supplementary movie.] From each crystal, a superset of 60 consecutive images were collected while rotating about 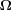 using a 0.5 or 1° oscillation range. *XDS* was used to auto-index these supersets and provide a benchmark orientation. The orientation was then determined using *XDS* auto-indexing from a smaller number of consecutive images. For each set of a given number of consecutive images, five subsets were randomly selected. The RC method was then used to compare all five orientation matrices with the benchmark matrix, and to calculate the difference between the observed and expected rotation. The data collections were done using only the well characterized and precise 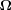 axis on the MD2m diffractometer at ID14-4 (McCarthy *et al.*, 2009 ▸). The data were collected at 12.7 keV using a beam size of 100 × 100 µm (horizontal × vertical) to ensure the full coverage of the crystal on an ADSC Q315r detector with a closed miniKappa (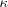 = 0) configuration. Experiments were performed at 100 K on flash frozen crystals with a mosaicity < 0.5°, as reported from the reference data set by *XDS*. Hence, all deviations in angular orientation resulted purely from the uncertainty of the indexing applied, including mosaicity as well as the limitations of the experimental observations. To determine the minimal requirements for useful experimental observations the statistics on the indexing stability were calculated using two to ten consecutive images. The angular differences between the supersets and the corresponding five randomly selected subsets are shown in Fig. 3 ▸.

While the variance is high when using only two consecutive images, we found that three consecutive images spanning 1° each were sufficient to reliably construct the orientation matrix; the inclusion of additional data increased the consistency of matrix determination, but the benefits were marginal.

### 3.2. Stability of rotation vector determination   

The precision of the direction vector determination of the rotation axes depends on how well the orientation (before and after the rotation) can be calculated as well as the mechanical accuracy of the goniometer movement itself. Hence, it is important to characterize each rotation axis in greater detail, and to perform a more complete and uniform sampling of the rotational space.

Thaumatin crystals (Nanao *et al.*, 2005 ▸) were used in omega scans on ID14-4 at the ESRF to collect five consecutive images at different orientations with starting goniometer settings of ω–κ–φ at various combinations of 0, 24, 148 and 240°. Hence, rotational difference could be compared not only with the zero setting but also between any of the settings applied. For a better characterization of the miniKappa goniometer head (Fig. 1 ▸), an extended list of rotational path differences was investigated for both kappa and phi motors. Next to the rotational differences, the corresponding angular positions used during the experimental observations are also provided in brackets: 24° (between 0 and 24°), 92° (between 148 and 240°), 124° (between 24 and 148°), 148° (between 0 and 148°), 216° (between 24 and 240°) and 240° (between 0 and 240°). For comparison, oscillation ranges were chosen as 0.1 and 1° for all data sets collected from two different crystals. All data sets were processed using *XDS* to determine the orientation matrix for each individually. The RC between each data set, differing in only one goniometer setting, was then applied to calculate a direction vector for the given rotation axis. The statistical angular differences between the mean of all calculated direction vectors of a given axis and the individual vectors have been determined and are shown in Table 1 ▸.

While outliers and large angular errors could indicate a problem with the hardware, a uniform distribution of small deviations, as in case of the miniKappa goniometer head used for this experiment, shows that a single discrete rotation about an axis is sufficient for properly characterizing its direction vector during routine calibrations (*e.g.* after unmounting and remounting, or during regular maintenance checks).

While the calculated direction vectors can be used for reorientation calculations, the agreement between the calculated and expected rotation angle for each axis provides information regarding the angular accuracy and precision of each motor. These statistics returned during rotation calibration are also useful in troubleshooting and provide complementary information to that gained during a translation calibration as previously discussed by Brockhauser *et al.* (2011 ▸).

Three primary factors affect sample orientation and location accuracy: the sphere of confusion, angular accuracy and goniometry precision (Davis *et al.*, 1968 ▸). The sphere of confusion refers to the minimum spherical volume traced by the movement of an infinitesimally small sample rotated in full about each axis under ideal conditions. This topic is outside the scope of this paper, as it is primarily determined by the goniostat design. The latter topics are of interest here as they can be characterized and tuned at the instrument control level. They directly affect the reorientation of the crystal, and ignoring either can lead to a failure of automated data collection and analysis. As such, checking for errors related to these effects while calibrating the goniometer and determining the directions of the rotation axes is of great importance. Analysis of goniometer rotation axis misalignment has been addressed before (Paciorek *et al.*, 1999 ▸; Schiltz & Bricogne, 2009 ▸; Brockhauser *et al.*, 2011 ▸).

Both the angular accuracy and goniometer precision are critical in reducing a data set to interpretable information. The orientation matrix 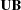 for a crystal of known unit-cell parameters provides an excellent standard from which to essentially back-calculate such information. The 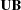 matrix describes the orientation of a crystal’s symmetry-defined coordinate system relative to the experimental coordinate system of the goniometer setup. For a calibration protocol, the question relates to the amount of data needed to reliably determine this matrix *via* the indexing of diffraction patterns. The use of automatic indexing in programs such as *MOSFLM*, *DENZO*, *XDS* and *DIALS* (Leslie, 2006 ▸; Otwinowski & Minor, 1997 ▸; Kabsch, 1988 ▸, 2010 ▸; Waterman *et al.*, 2016 ▸) makes such a protocol easy to perform and integrate in beamline maintenance.

## 4. Conclusions   

This study illustrates the ease with which the rotation calibration of a multi-axis goniometer system can be performed. Such a procedure for all axes of a kappa goniometer can be carried out on the beamline in a matter of minutes, as a minimum of only four diffraction images from a well diffracting test sample need to be collected in order to provide an insight into the accuracy and precision of the rotational aspects of a diffractometer setup.

The described rotation calibration together with the translation calibration (Brockhauser *et al.*, 2011 ▸) is implemented by the software *STAC* (Brockhauser *et al.*, 2013 ▸) to support precise crystal reorientations as integrated into the *EDNA* framework (Incardona *et al.*, 2009 ▸) and to support the automation of complex experimental protocols (Brockhauser *et al.*, 2012 ▸).

## Supplementary Material

Supporting information file. DOI: 10.1107/S1600576718010956/ap5028sup1.mp4

## Figures and Tables

**Table 1:** Average angular error in rotation axis direction calculated from two thaumatin data sets For each set, five images were collected with (ω, κ, φ) at different combinations of [0°, 24°, 148°, 240°], such that the majority of rotational space was sampled. Each set was processed using XDS. Rotation axis direction vectors 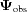 for each orientation matrix, the angle between each direction vector for a given axis and the mean of all direction vectors for a given axis were calculated. Note that in the case of a single observation no variance is shown.

	Average angular error	
Axis	0.1° wedges	1.0° wedges
ω	0.20° ± 0.09	0.09° ± 0.01
24°	0.28°	0.07°
148°	0.10°	0.09°
240°	0.21°	0.11°

κ	0.15° ± 0.12	0.15° ± 0.06
24°	0.19°	0.20° ± 0.09
92°	0.12° ± 0.12	0.15° ± 0.10
124°	0.11° ± 0.05	0.11° ± 0.05
148°	0.13° ± 0.11	0.13° ± 0.05
216°	0.19° ± 0.13	0.16° ± 0.04
240°	0.23° ± 0.24	0.17° ± 0.02

φ	0.12° ± 0.06	0.07° ± 0.07
24°	0.24° ± 0.08	0.19° ± 0.10
92°	0.15° ± 0.03	0.08° ± 0.04
124°	0.12° ± 0.05	0.06° ± 0.03
148°	0.09° ± 0.05	0.03° ± 0.01
216°	0.11° ± 0.06	0.04° ± 0.02
240°	0.14° ± 0.11	0.08° ± 0.06
